# Top-down and middle-down proteomic analysis of Shiga toxin using MALDI-TOF-TOF mass spectrometry

**DOI:** 10.1016/j.mex.2019.04.011

**Published:** 2019-04-16

**Authors:** Clifton K. Fagerquist, William J. Zaragoza

**Affiliations:** Produce Safety & Microbiology, Western Regional Research Center, Agricultural Research Service, U.S. Department of Agriculture, 800 Buchanan Street, Albany, CA 94710, United States

**Keywords:** Top-down of Shiga toxin, Top-down proteomics, Mass spectrometry, STEC, Shiga toxin, MALDI-TOF-TOF

## Abstract

The method describes a step-by-step process for analysis of putative Shiga toxin-producing *Escherichia coli* (STEC) for expression of Shiga toxin (Stx). The technique utilizes antibiotic induction, mass spectrometry and top-down/middle-down proteomic analysis. Stx expression is induced by overnight culturing of a STEC strain on Luria-Bertani agar (LBA) supplemented with DNA-damaging antibiotics. Culturing on agar media avoids sample contamination from salts, small molecules, peptides, etc. present in broth media that would interfere with protein ionization by matrix-assisted laser desorption/ionization (MALDI). No mechanical lysis of bacterial cells is required to release the toxin as the antibiotic triggers the lytic cycle of the bacteriophage resulting in toxin expression *and* bacterial cell lysis. Unfractionated samples are analyzed by MALDI-time-of-flight-time-of-flight (MALDI-TOF-TOF) mass spectrometry and tandem mass spectrometry (MS/MS) using post-source decay (PSD). New features of the method are the following.

• Each putative STEC strain is systematically screened for toxin expression using two different antibiotics at two different concentrations: ciprofloxacin at 10 and 20 ng mL^−1^ and mitomycin-C at 800 and 1200 ng mL^−1^ to determine the optimal antibiotic and concentration for toxin expression for each strain.
• The grid-to-source voltage of MALDI-TOF-TOF is optimized to maximize PSD efficiency.

## Graphical abstract

**Figure:**
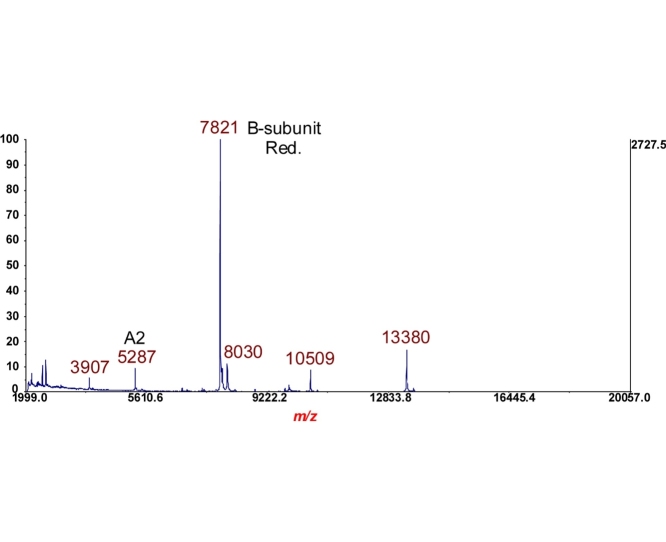

**Specifications Table**

**Table:** 

Subject Area:	Chemistry
More specific subject area:	Mass spectrometry-based proteomics of Shiga toxin types and subtypes
Method name:	Top-down of Shiga toxin
Name and reference of original method:	Fagerquist CK, Zaragoza WJ. Bacteriophage cell lysis of Shiga toxin-producing Escherichia coli for top-down proteomic identification of Shiga toxins 1 & 2 using matrix-assisted laser desorption/ionization tandem time-of-flight mass spectrometry. Rapid Commun Mass Spectrom. 2016 Mar 30;30(6):671-80.
Resource availability:	USDA top-down proteomic software is available free of charge to non-profit research institutions after completion of a material transfer agreement.

## Method details

### Bacterial culturing and sample preparation

1 Handling and manipulation of Shiga toxin-producing *Escherichia coli* (STEC) should be performed in a Class-2 Biohazard safety cabinet (BSL-2).
2 STEC were cultured overnight on Luria-Bertani agar (LBA) supplemented with antibiotic. As each STEC strain may respond differently to antibiotic exposure, we initially screened each strain for optimal expression of Shiga toxin (Stx) using two different concentrations of ciprofloxacin (10 and 20 ng mL^−1^) and mitomycin-C (800 and 1200 ng mL^−1^). STECs were also cultured on LBA without antibiotic to assess protein expression in unchallenged cells.
3 Detection of Stx during this initial screening process was performed in MS linear-mode for the disulfide bond-intact B-subunit as its mass, secondary structure and stoichiometric abundance (AB_5_) has a high probability for detection by MALDI.
4 Once the optimal antibiotic (and concentration) was found for a STEC strain, it was used in subsequent sample preparation and analysis steps. Typically, the optimal antibiotic concentration allowed for sufficient bacterial growth to fill (at least) a flush 1 μL loop.
5 A flush 1 μL loop of cells was transferred to a sterile screw-cap microcentrifuge tube containing 300 μL of water (Fisher Chemical). A flush loop consists of filling the hole of the loop completely but not beyond the depth (or thickness) of the loop.
6 The tube was briefly vortexed (<15 s) to disperse cellular and extracellular material in solution but avoid, as much as possible, cell lysis [1]. This is in contrast with our previous approach that utilized bead-beating for lysis [[2], [3], [4]]. The current approach relies upon antibiotic induction for *both* expression of Stx and cell lysis via antibiotic triggering the λ bacteriophage lytic cycle.
7 After brief vortexing, the tube was centrifuged at 13,000 rpm for 5 min to pellet cellular material.
8 For analysis of the *disulfide bond-intact* B-subunit (lasso-loop secondary structure), a 0.6 μL aliquot of the supernatant is spotted directly onto the 384-spot stainless steel MALDI target plate without further sample preparation and allowed to dry. The dried sample spot is then overlayed with a 0.6 μL aliquot of saturated solution (33% acetonitrile, 67% water and 0.1% trifluoracetic acid) of sinapinic acid MALDI matrix which is also allowed to dry. It is recommended that the matrix solution be made fresh for each day’s experiment.
9 Step 8 is performed in a BSL-2 biosafety cabinet. The high rpm centrifugation of the sample for 5 min (Step 7) should pellet any cells allowing cell-free extraction of the 0.6 μL aliquot of supernatant. However, the supernatant may be filter sterilized with a 0.2 μm filter as an extra precaution.
10 Sample and matrix spotting/drying can produce variable results at times due to laboratory humidity and other factors. In consequence, a *minimum* of five spots (five technical replicates) per sample should be performed.
11 For analysis of the *disulfide bond-reduced* B-subunit (linear chain secondary structure), 0.5 μL of 1 M dithiothreitol (DTT, Sigma-Aldrich) is added to 30 μL of supernatant and is heated in a water bath at 70 °C for 10 min in an Eppendorf tube. A 0.6 μL aliquot of this solution is spotted directly onto the stainless steel MALDI target plate and allowed to dry. The dried sample spot is then overlayed with a 0.6 μL aliquot of saturated solution of sinapinic acid MALDI matrix which is also allowed to dry.
12 For analysis of *both* the A2 fragment of the A-subunit and the disulfide bond-reduced B-subunit, 0.5 μL of furin (New England Biolabs, MA) is added to 30 μL of supernatant in an Eppendorf tube, incubated at 37 °C for 45 min after which 0.5 μL of DTT is added to the tube and the solution is heated in a water bath at 70 °C for 10 min. A 0.6 μL aliquot of this solution is spotted directly onto the stainless steel MALDI target plate and allowed to dry.
13 Caveat: Furin is shipped by the vendor on dry ice and in a solution that contains glycerol so that it remains cold but does not freeze and damage the enzymatic activity of the furin. However, glycerol is viscous compound and does not evaporate readily at room temperature. The glycerol present in the furin-treated sample will not dry completely (or at all) when spotted onto the MALDI target. After spotting, allow 15 min for the spot to “dry”.
14 The “dried” furin-treated sample spot is then overlayed with a 0.6 μL aliquot of saturated solution of sinapinic acid MALDI matrix which is also allowed to dry (and gives the appearance of drying).

### Mass spectrometry

**Fig. 1:**
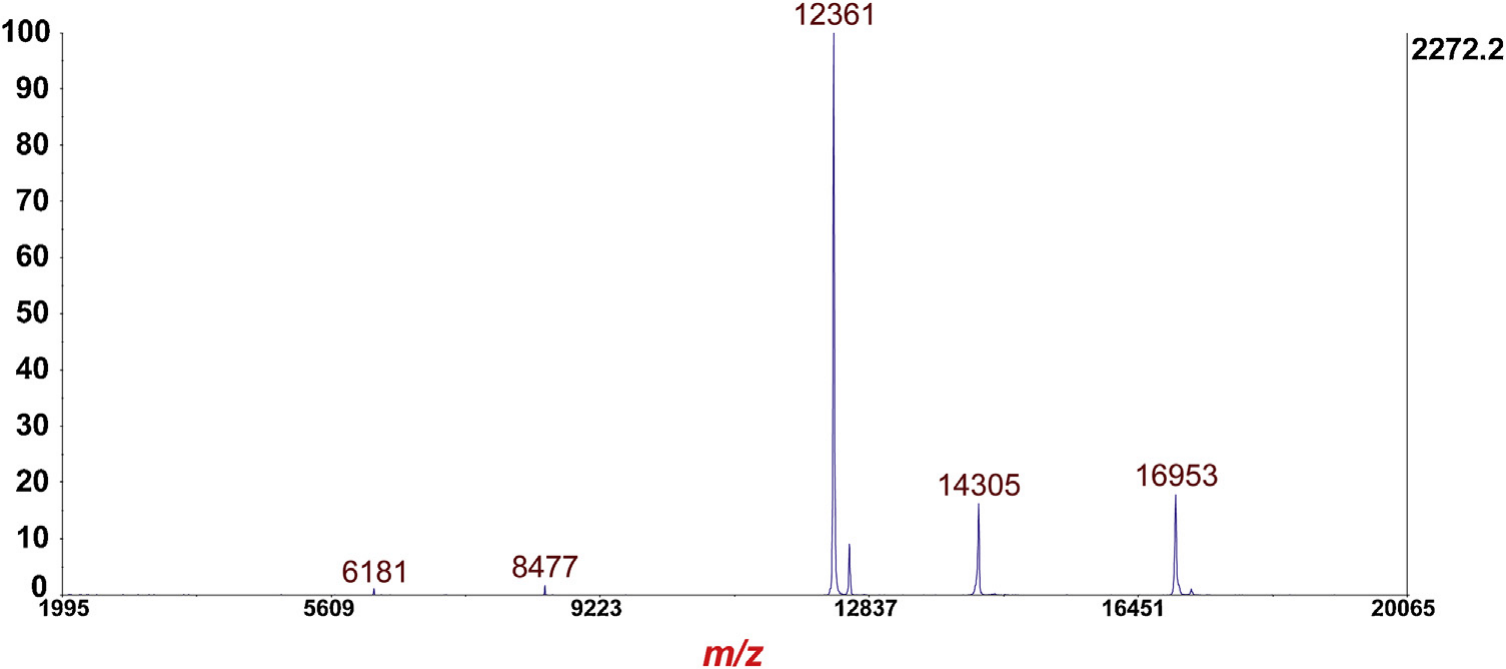
Calibration of MS linear-mode is performed using the +1 and +2 charge states of cytochrome-C (Ave. mass: 12,360 Da), myoglobin (Ave mass: 16,952 Da) and lysozyme (Ave mass: 14,305 Da). Minimum of five ions to match for calibration.

**Fig. 2:**
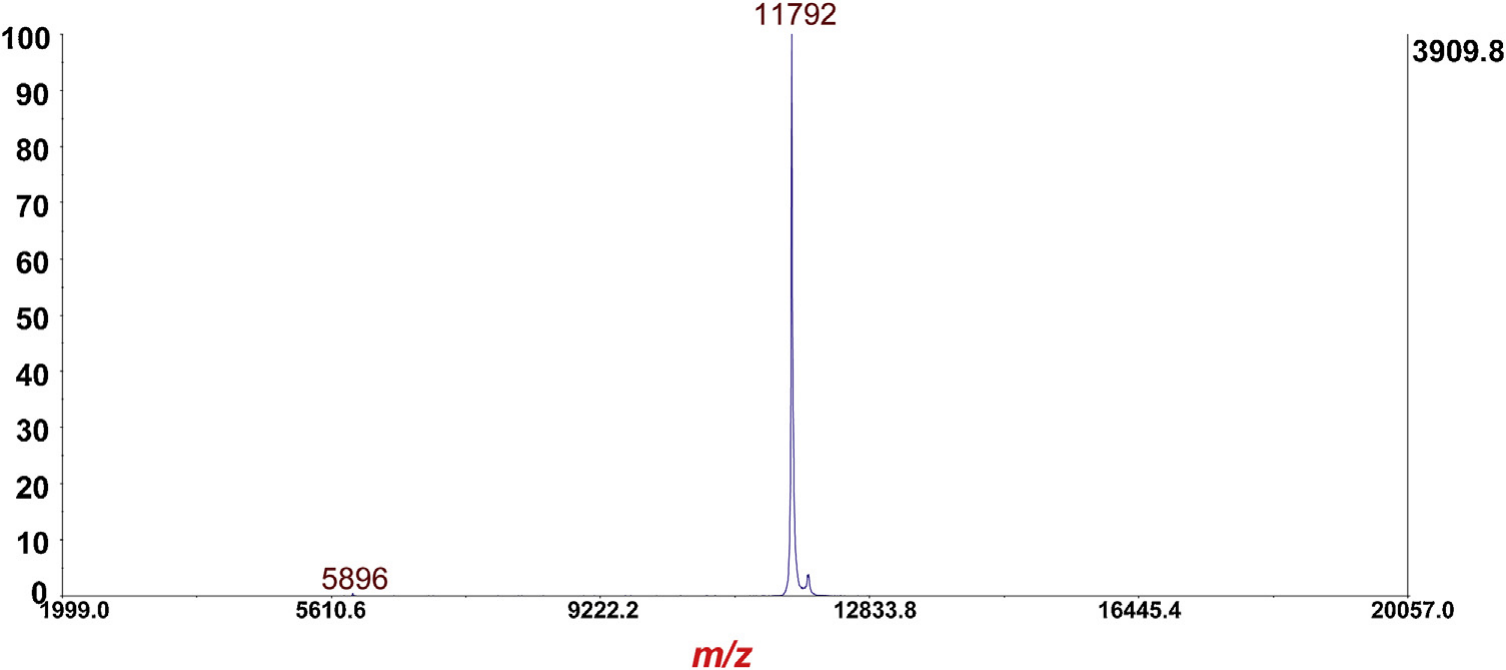
Analysis of alkylated thioredoxin (calibrant for MS/MS mode) in MS linear-mode (Ave mass: 11,789.6 Da [5].

1 After spots have dried and before inserting the MALDI plate into the mass spectrometer, its surface should be blown with high purity dry nitrogen gas (ten to fifteen seconds at a pressure of 25 psi). House nitrogen is usually adequate if it is from the headspace of a liquid nitrogen Dewar. The dry nitrogen is to remove any particulate material that could be transferred to the ion optics when the plate is inserted into the mass spectrometer.
2 The plate is then inserted into a 4800 MALDI-TOF-TOF mass spectrometer (*Sciex*, Redwood City) equipped with a 355 nm solid-state pulsed YAG laser (200 Hz repetition rate, 5 nsec pulse width).
3 Once plate insertion and pump down is completed, test ionization/detection of the linear-mode protein calibrants (cytochrome-C, lysozyme, myoglobin) in linear mode (20.0 kV). Although the pressure in Source 1 is usually still too high, protein calibrants should be detectable. If protein ion signal is obtained, wait 30–45 min for power supplies to warm-up and stabilize as well as allow time for the pressure in Source 1 to drop further. Do not wait too long to resume analysis as the laser and power supplies may turn off.
4 Acquire data for calibration using mid-mass linear-mode which has a mass-to-charge (*m*/*z*) range of 2000 Th to 20,000 Th with a focus mass of 9000 Da.
5 The +1 and +2 charge states of the protein calibrants: cytochrome-C, myoglobin and lysozyme are used for calibration.
6 It was determined that highly purified (re-crystallized) sinapinic acid MALDI matrix was found to be the best matrix for post-source decay (PSD) of +1 protein ions when coupled with high laser fluence using a pulsed 355 nm solid-state (YAG) laser. However, unlike α-cyano-cinnamic acid matrix (CCA), sinapinic acid does not result in highly abundant +2 charge state protein ions (Fig. 1). As it is desirable to use both +1 and +2 protein ions for linear-mode calibration, it is important to use a laser fluence just above *threshold* to obtain both the +1 and +2 charge states of these protein ions.
7 *Threshold* is defined here as that amount of laser intensity necessary to see a detectable analyte signal. This level of laser fluence will also provide the highest resolution and mass accuracy as space charge effects are minimal. If +2 charge states are not detected try the other spots. Matrix crystallization with the embedded analyte is not uniform and that is why multiple spotting is useful. Paradoxically, if you attempt to “boost” the signal of the +2 charge state proteins ions by increased laser fluence, the +2 charge states may disappear altogether because of increased collisions with the higher density of matrix molecules in the matrix plume. Solution: dial back the laser intensity to detect more of the +2 protein ions. Preferably, one should obtain a minimum of five peaks for calibration after which the calibration file in the operating mode is updated and then closed.
8 The 4800 MALDI-TOF-TOF has a MS linear-mode external calibration specification of 1000 ppm. For example, for a protein of molecular weight of 10,000 Da, the mass accuracy with external calibration is 10,000 ± 10 Da. We have found the mass accuracy to be usually better than the stated specification.
9 Before proceeding with sample acquisition, collect MS linear data on the calibrant to be used for MS/MS-PSD: alkylated thioredoxin (AlkTrx) [5]. Collect 1000 laser shots (5 s) of a AlkTrx spot and save the data file. Average *m*/*z* of [M+H]^+^ should be around 11,790 Th (Fig. 2).
10 Proceed with sample(s) acquisition by collecting 1000 laser shots (5 s acquisition) of each spot of interest. The laser power/intensity can be adjusted higher if insufficient signal is obtained although try other spots if one spot does not produce sufficient signal.
11 Once reasonable data is obtained save the file and print out a hard copy for reference for collecting data in MS/MS mode.
12 Complete all MS linear-mode data collection before collecting data in MS/MS mode.
13 Open the acquisition method of MS/MS reflectron-mode 1 kV sensitivity and set as active. The 1 kV sensitivity MS/MS mode typically gave us the best fragmentation results of protein ions. No collision gas is introduced into the collision cell, and the metastable suppressor is ON. In consequence, fragmentation is a result of PSD. Although we had tried using high energy collision induced dissociation (HE-CID), we found that for protein ions little advantage in using a collision gas as any extra fragmentation that may be obtained was offset by a loss of sensitivity due to ion scattering.
14 It should be noted that in MS/MS mode ions are accelerated from the 1st source at 8 kV, decelerated to 1 kV in the collision cell and re-accelerated to 15 kV in the 2nd source. Ions are then reflected in the two-stage reflectron where they are turned nearly 180° which extends the length of their flight path and subsequently striking the reflectron detector.
15 Another acquisition method is MS/MS reflectron-mode 2 kV sensitivity. The acquisition method is worth trying if results with 1 kV sensitivity are not adequate.
16 As with MS linear-mode, MS/MS reflectron-mode needs to be calibrated. The MS/MS-PSD sequence-specific fragment ions of AlkTrx are used to calibrate reflectron-mode as shown in Fig. 3 [5].
17 In order to achieve efficient PSD, the laser fluence is increased significantly above threshold to such an extent that ablation of the sample/matrix spot is visible during laser firing. Ten thousand (10,000) laser shots (50 s acquisition) are acquired/summed for each MS/MS-PSD experiment after which there is little sample/matrix of the spot remaining.
18 Two critical operator-controlled parameters for a MS/MS experiment is precursor ion *m*/*z* and the setting of the timed-ion-selector or TIS. The precursor ion *m*/*z* can be obtained from the previously acquired MS linear-mode data of the sample, i.e. the *m*/*z* of the target ion to be fragmented. The TIS parameter allows adjustment of the width of the isolation window used to permit the target ion to pass into the collision cell and exclude all other ions. The TIS is a velocity analyzer or gate, i.e. ions are allowed pass or not pass based on their *arrival time* at the TIS. Only ions that arrive at the right time are allowed through the TIS. Ion velocity is calculated by υ = (2 eV/m)^1/2^ and thus dependent on the inverse square root of the mass of the ion. Thus, ions of different mass will have the same kinetic energy (eV) but different velocities.
19 The TIS window need be no wider than ±100 Da for proteins under 10 kDa. If there are abundant ions in proximity to the target ion (<100 Da), the TIS can be narrowed on either side (or both sides) to as little as 15 Da. This narrowing of the TIS will reduce the intensity of the target ion but will exclude non-target ions from entering the collision cell and contaminating the MS/MS experiment with non-target fragment ions.

### Processing of mass spectrometry data

1 MS data was either not processed or a Noise Filter (Correlation Factor: 0.7) was applied.
2 MS/MS data was processed in the following sequence of steps:
  a Advanced Baseline Correction (Peak Width: 32; Flexibility: 0.5; Degree: 0.1)
  b Noise Removal (2 standard deviations to remove)
  c Gaussian Smooth (Filter Width: 31 points)
3 For top-down and middle-down proteomic analysis, the MS/MS data was further centroided and the ASCII Spectrum was exported from Data Explorer software (Sciex) as text file of fragment ions of *m*/*z* and absolute intensity.
4 The ASCII file was uploaded to the USDA top-down proteomic software [6,7].

### Top-down and middle-down proteomic analysis

1 The USDA top-down proteomic software has been described in detail [6,7]. The software is available to other researchers as outlined in the Acknowledgments.
2 Protein identifications were based on a comparison of the *m*/*z* of fragment ions in the MS/MS of the unknown protein to a database of in silico fragment ions (a, b, y, b-H_2_0, y-H_2_0, y-NH_3_) of thousands of bacterial protein sequences having the same mass as the unknown protein within a pre-set tolerance that is under operator control.
3 The operator can also set the minimum peak intensity threshold (%) of the MS/MS fragment ions depending the quality of the MS/MS data. High quality MS/MS data have less noise and a lower noise threshold can be used.
4 The software also allows the operator to compare to *all* in silico fragment ions of a protein sequence or only those fragment ions adjacent to specific residues. For example, since it is known experimentally that metastable protein ions have a greater propensity to fragment on the C-terminal side of aspartic acid (D) and glutamic acid (E) residues and the N-terminal side proline (P) residues, the MS/MS-to-in silico comparison can be restricted by the operator to compare only those in silico fragment ions that are adjacent to specific residues, e.g. D, E and P.
5 The software scores and ranks MS/MS-to-in silico comparisons using both a P-value scoring algorithm [6,8] and the % of matched MS/MS fragment ions [6,7].
6 Another USDA-developed software program is used to generate in silico fragment ion files which are uploaded to the in silico database [2].

## Method validation

### Calibration MS linear-mode

### Calibration MS/MS reflectron-mode

**Fig. 3:**
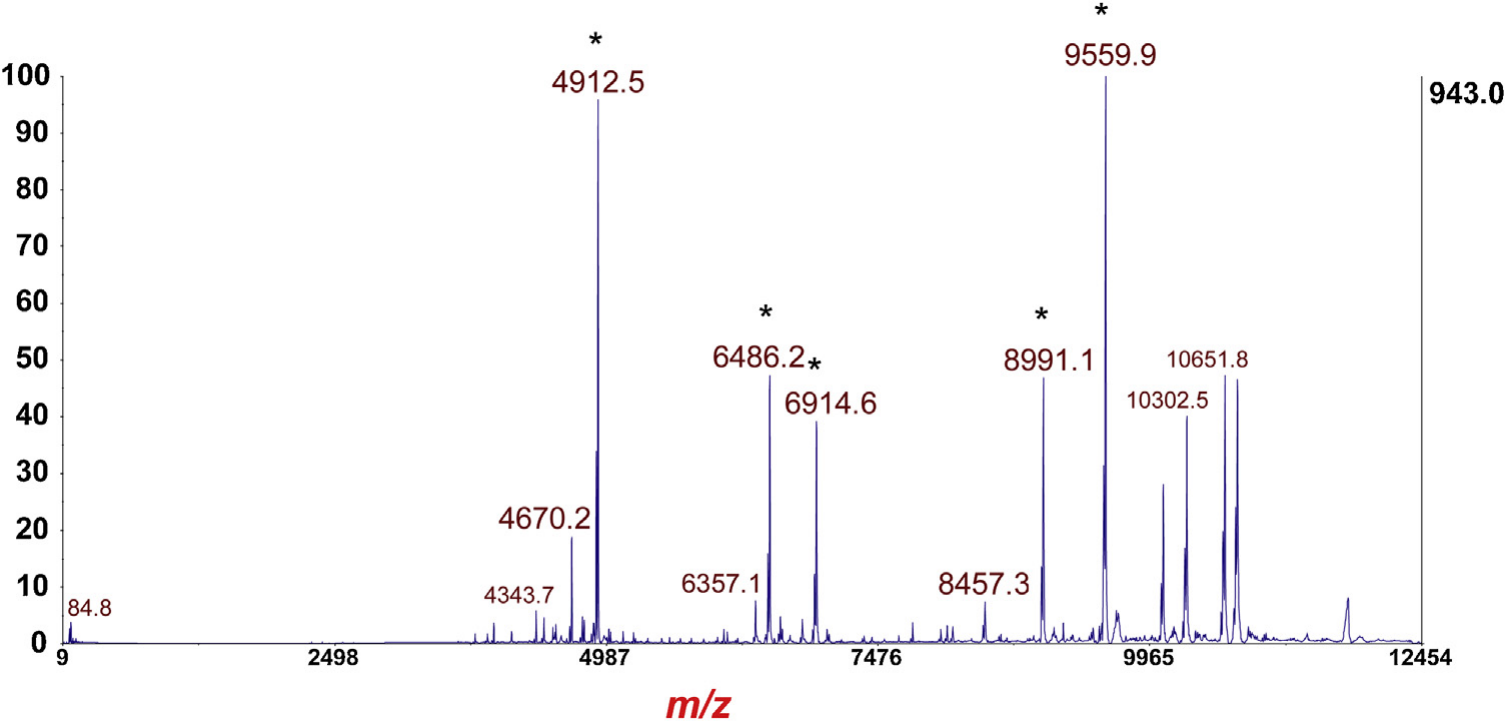
MS/MS of *m*/*z* 11,792 [M+H]^+^ alkylated thioredoxin in Fig. 2. Fragment ions marked with asterisk are used for calibration of the MS/MS reflectron-mode.

### The effect of laser fluence intensity and *Grid-to-Source 1* voltage ratio on the efficiency of post-source decay (PSD) of protein ions

Post-source decay (PSD) of protein ions using MALDI-TOF or MALDI-TOF-TOF instruments have been observed and utilized for decades PSD is a low energy dissociation technique that has found utility for certain applications. It was observed that PSD of protein ions was found to be more efficient when using a laser fluence significantly higher than that necessary for protein ionization, i.e. significantly above ionization *threshold*. Typically, the best performance of MALDI-TOF-MS is obtained when the laser fluence or intensity is adjusted to be slightly above threshold as this gives best resolution and mass accuracy because it minimizes space charge effects. For PSD, however, it was speculated that the higher laser intensity results in a greater number of matrix/analyte collisions that deposit energy into the analyte molecule leading to its subsequent dissociation after being accelerated from the source [8]. It should be clarified that, with the introduction of delayed ion extraction or pulsed ion extraction, the firing of the laser is not simultaneous with acceleration of the ions from the source. There is a delay (˜300 to 600 ns) between the laser pulse (˜5 ns pulse width) and the pulsed acceleration of ions from the source. Matrix/analyte collisions could occur during the laser firing as well as during the acceleration of ions from the source. Clearly a laser fluence significantly above threshold will ablate/exhaust the sample spot more quickly but also result in a greater density of matrix molecules in the plume of the narrow space through which the analyte ions must traverse when they are *accelerated* from the source. The voltage at which the ions are accelerated from the source will play a role in the energy deposition into analyte ions from energetic collisions with matrix molecules.

**Fig. 4:**
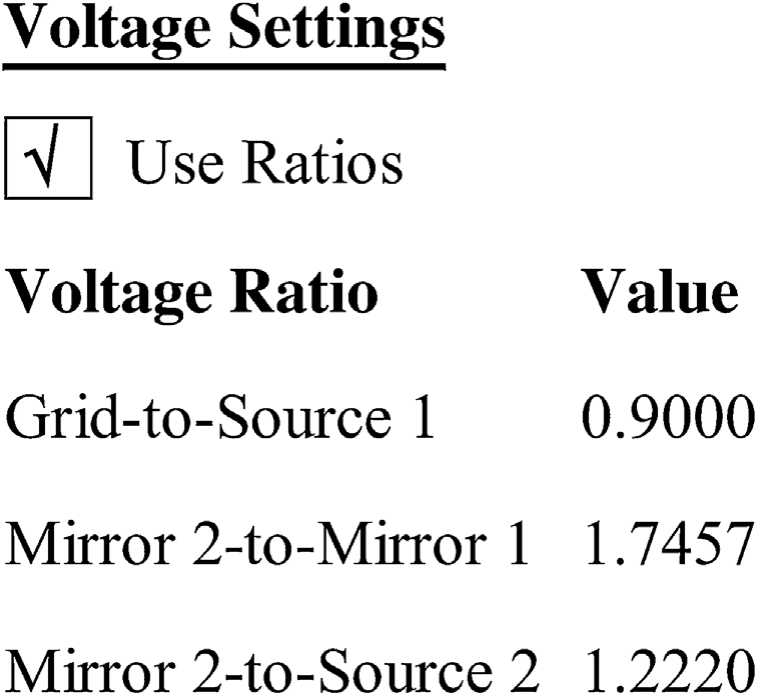
Adjusting *Grid-to-Source 1* ratio in operating mode of *MS-MS Positive Ion* acquisition method.

The ratio of the *Grid-to-Source 1* voltages was varied in the operating mode of the *MS-MS Positive Ion* acquisition (Fig. 4) to test its effect on the efficiency of MS/MS-PSD of the B-subunit of Stx2. “Source 1” is the stainless steel MALDI plate and the “Grid” is the funnel/cone-like ion optic positioned ˜2 mm directly above the MALDI plate. At a Grid-to-Source 1 ratio of 0.9000, the potential difference between these two elements is ˜800 V which is responsible for the initial acceleration of ions from that narrow spatial volume.

**Fig. 5:**
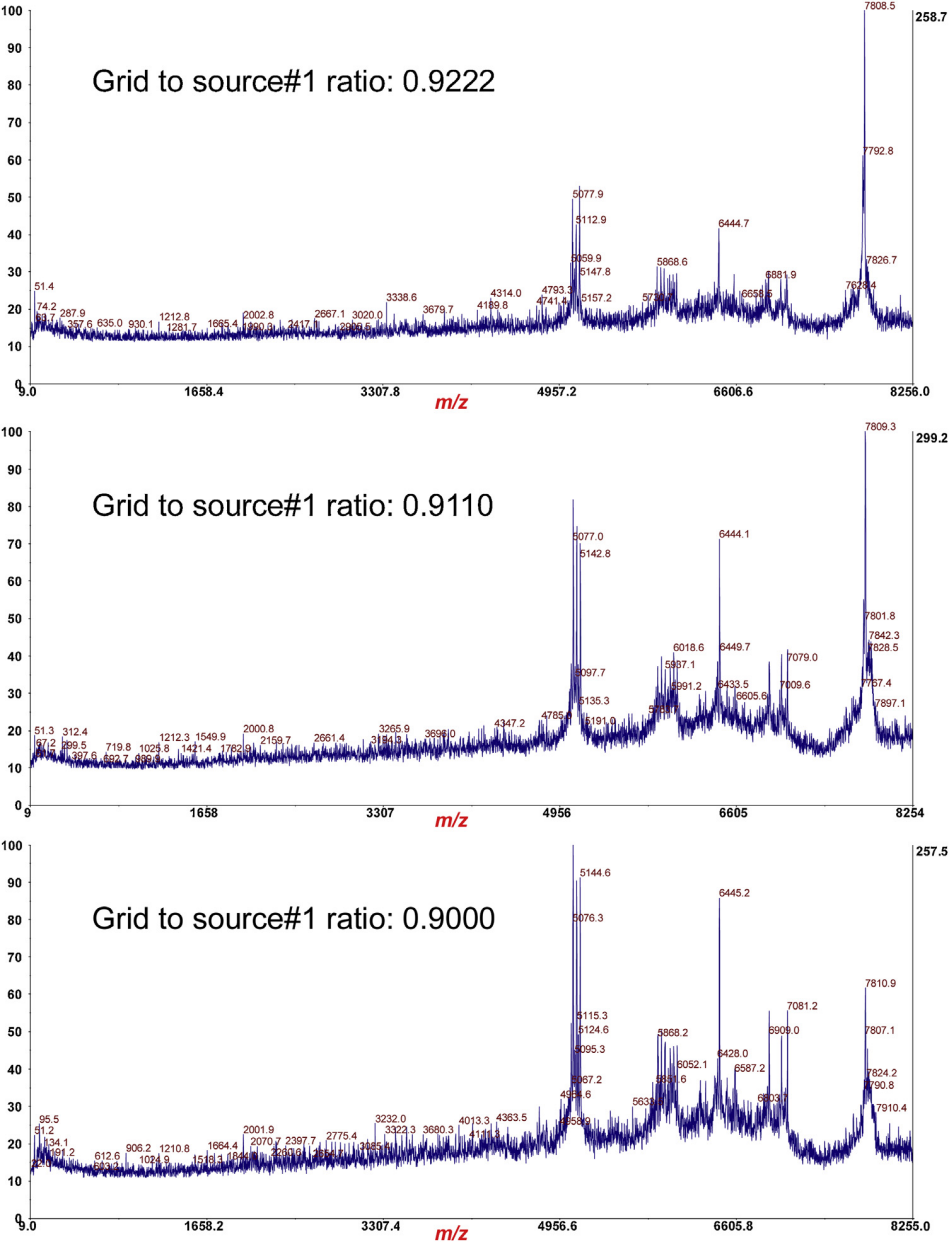
Effect of adjusting Grid-to-Source 1 ratio of MS/MS-PSD of B-subunit of Stx2.

As can be seen in Fig. 5, the greater potential difference (lower ratio) between Source 1 and the “Grid”, the greater amount of protein fragmentation that is observed suggesting that collisions between neutral matrix molecules and analyte ions during ion acceleration contribute to the efficiency of protein ion fragmentation after leaving the source, i.e. post-source decay.

It has also been observed that the +2 charge states of calibrant protein ions, which are always weak in intensity when using sinapinic acid MALDI matrix (Fig. 1), become nearly undetectable at high laser fluence due, presumably, to collisions with the high density of matrix molecules that can deflect these less intense ions from exiting the source.

### Analysis of positive control STEC strain

**Fig. 6:**
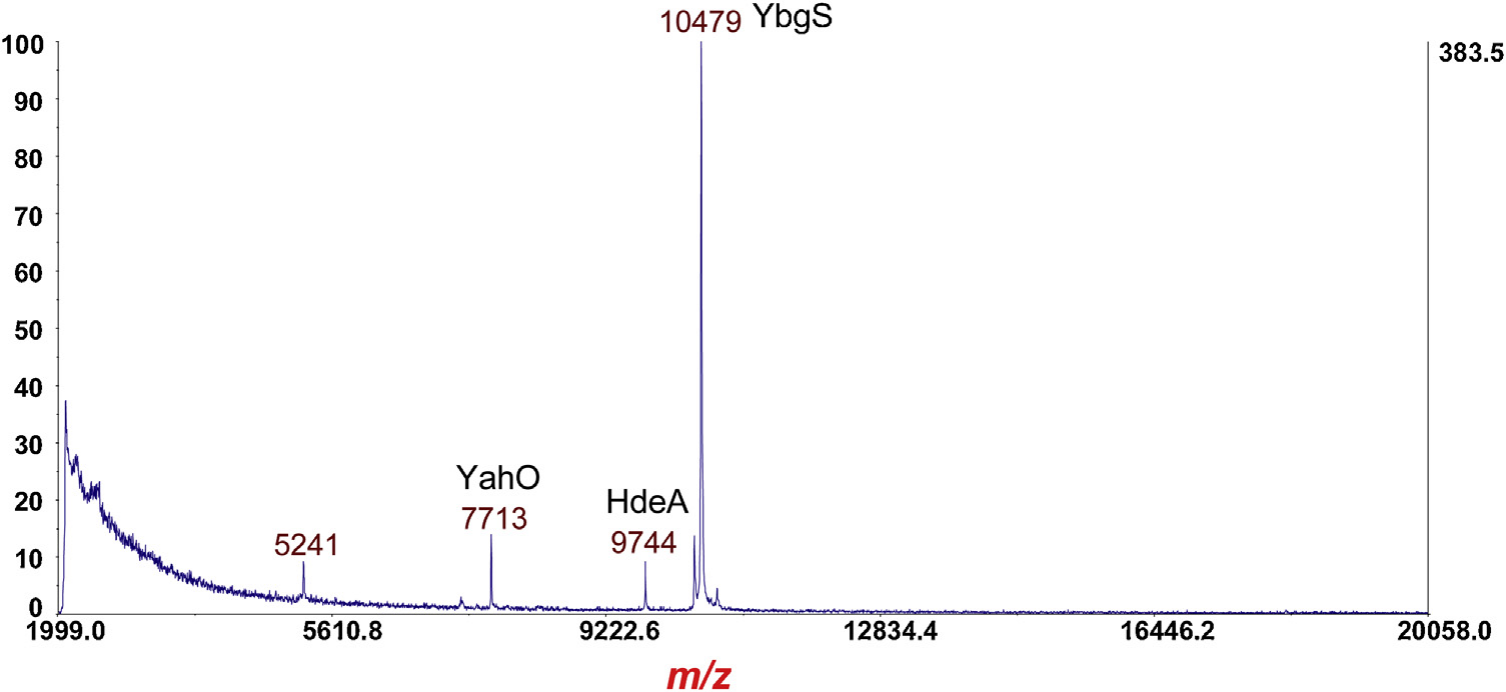
MS of *Escherichia coli* O157:H7 strain EDL933 cultured overnight on LBA. A few bacterial protein ions are detected even though there was no deliberate mechanical lysis of cells. These protein ions had been previously identified by top-down proteomic identification [9]. No Stx is detected under these culturing conditions.

**Fig. 7:**
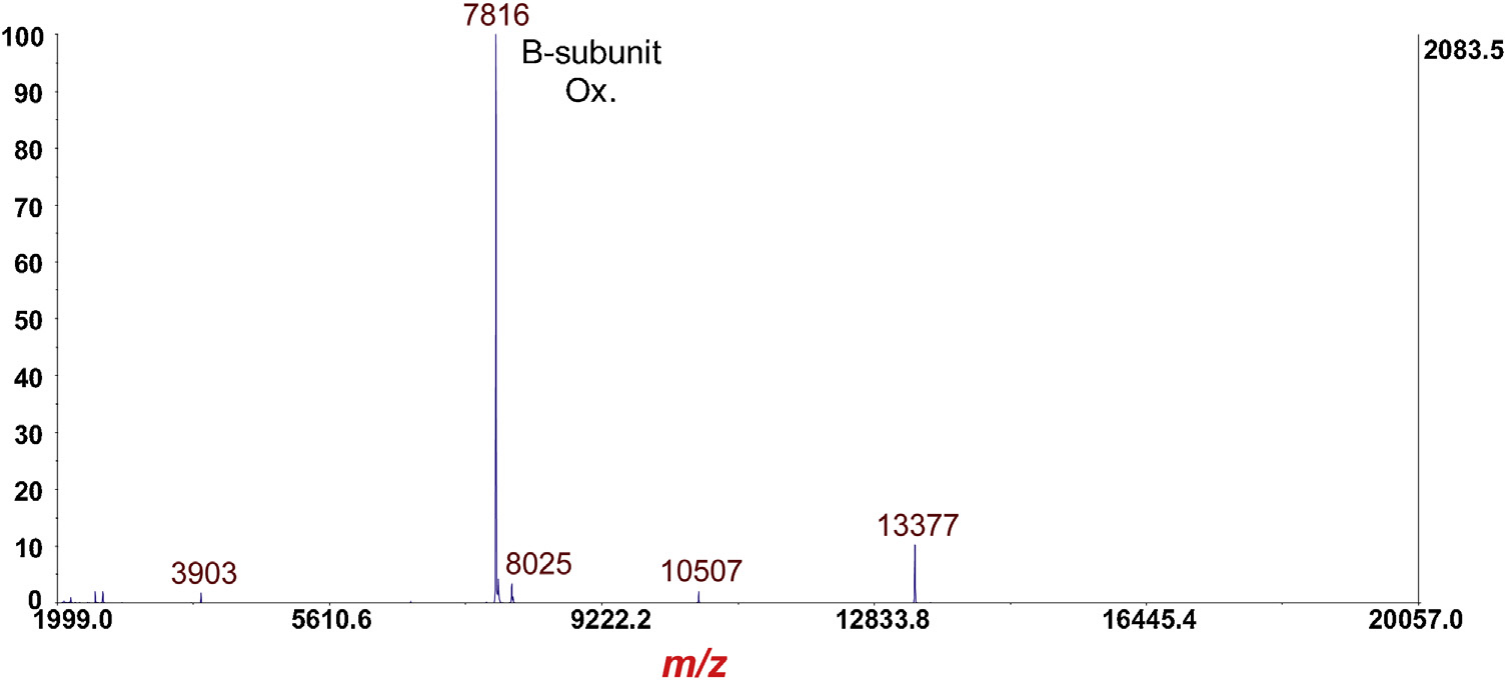
MS of *Escherichia coli* O157:H7 strain EDL933 cultured overnight on LBA supplemented with 20 ng/mL of ciprofloxacin. The prominent protein ion at *m*/*z* 7816 is the [M+H]^+^ of the B-subunit of Stx2a (disulfide bond intact). Phage related proteins are also detected at *m*/*z* 10,507 and 13,377.

**Fig. 8:**
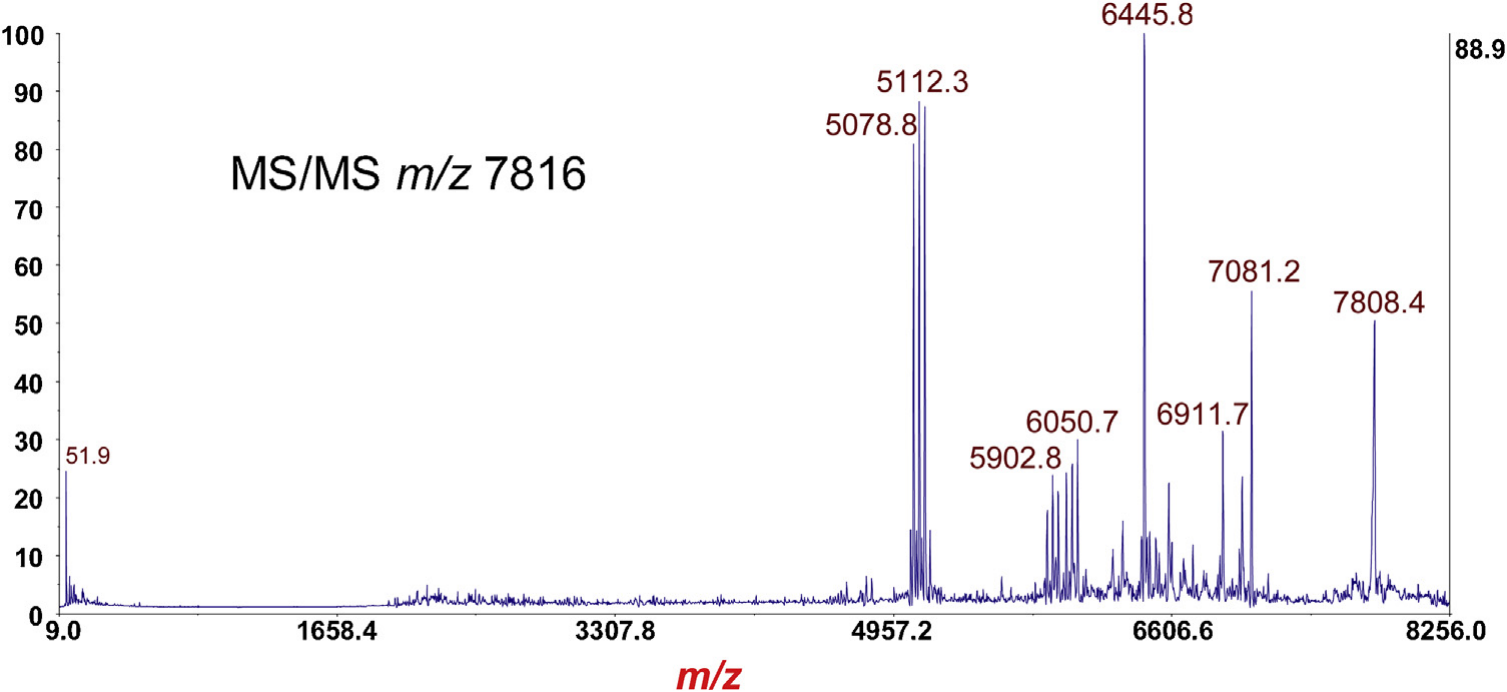
MS/MS-PSD of *m*/*z* at 7816 of the B-subunit from Fig. 7. Isolation mass window: ±100 Da. Note fragment ion triplets due to polypeptide backbone cleavage *between* the two cysteine residues of the disulfide bond *and* symmetric/asymmetric cleavage of the disulfide bond.

**Fig. 9:**
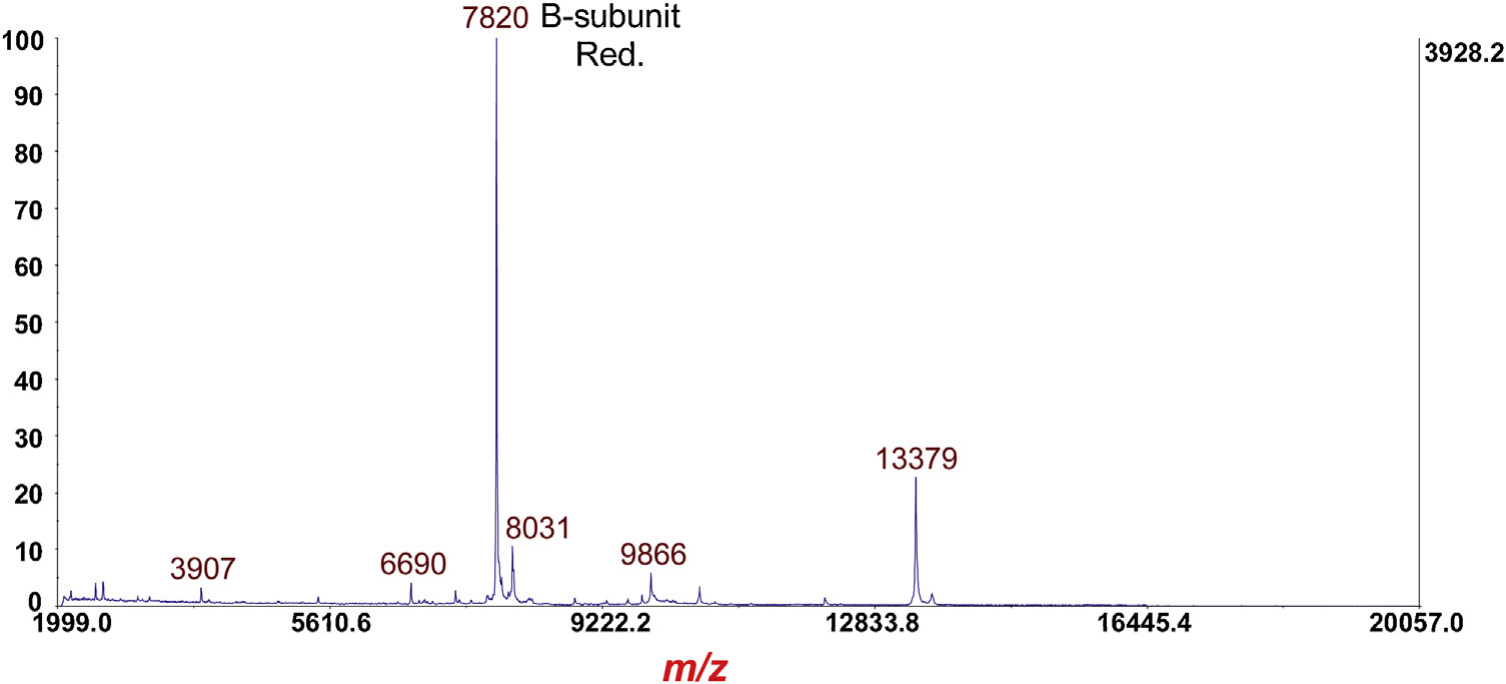
MS of *Escherichia coli* O157:H7 strain EDL933 cultured overnight on LBA supplemented with 20 ng/mL of ciprofloxacin followed by disulfide reduction with DTT. The prominent protein ion at *m*/*z* 7820 is the [M+H]^+^ of the B-subunit of Stx2a (disulfide bond reduced).

**Fig. 10:**
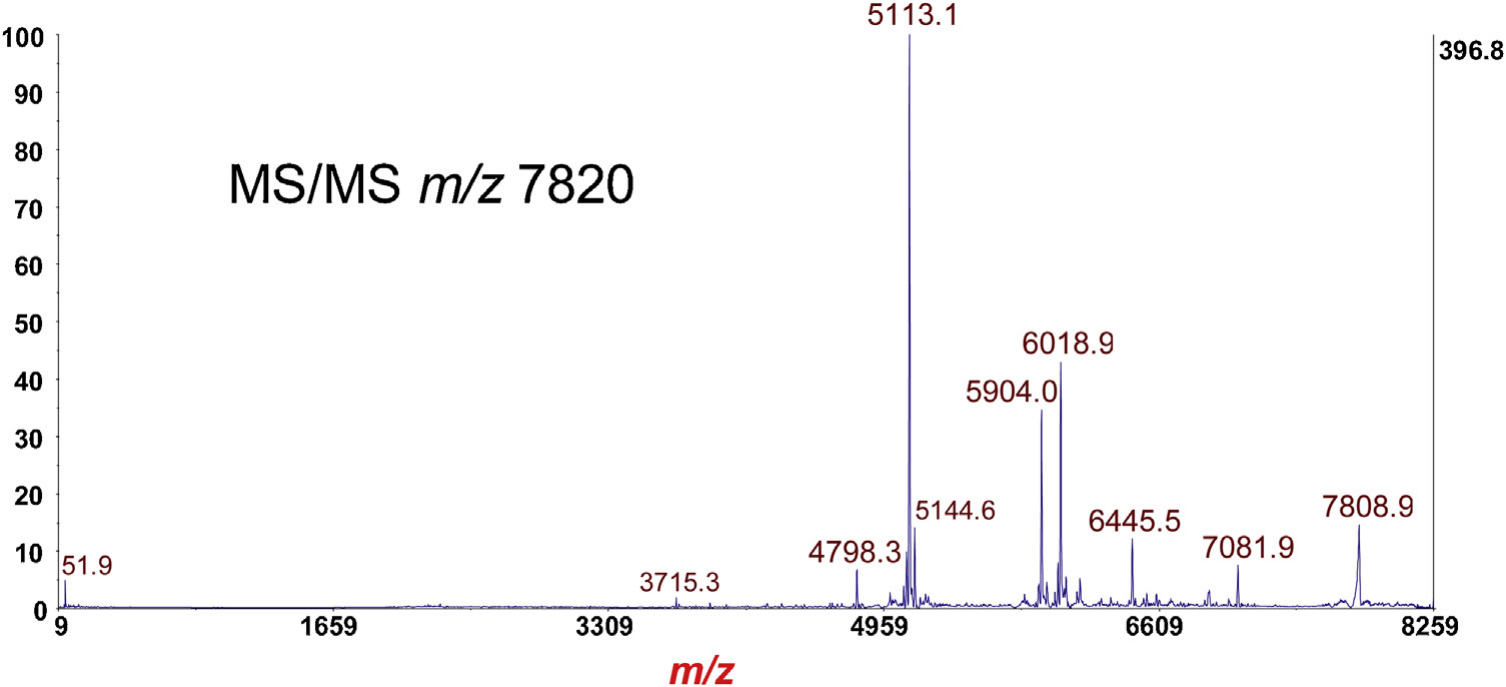
MS/MS-PSD of *m*/*z* at 7820 of the B-subunit (disulfide reduced) from Fig. 9. Isolation mass window: ±100 Da. Note the *absence* of fragment ion triplets due to reduction of the disulfide bond.

**Fig. 11:**
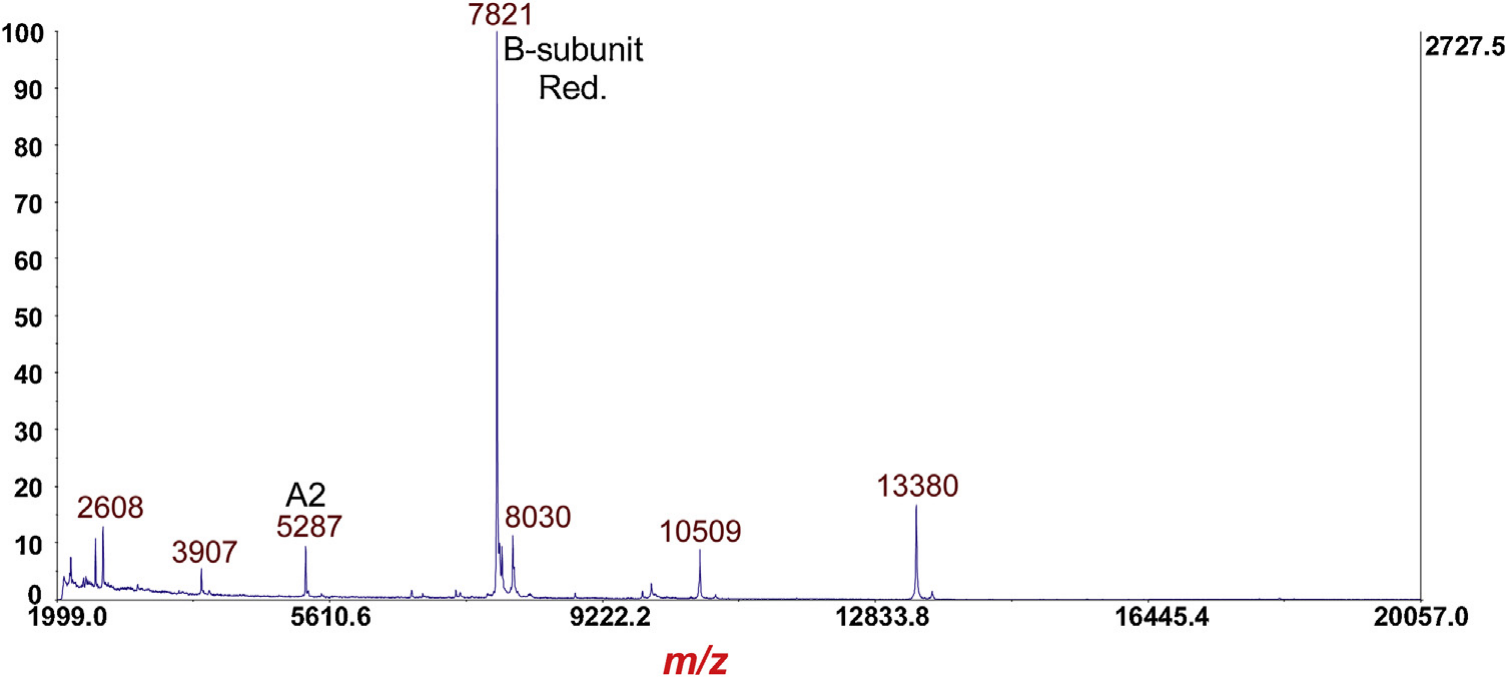
MS of *Escherichia coli* O157:H7 strain EDL933 cultured overnight on LBA supplemented with 20 ng/mL of ciprofloxacin followed by furin digestion and disulfide reduction with DTT. The protein ion at *m*/*z* 5287 is the [M+H]^+^ of the A2 fragment of the A-subunit of Stx2a. The reduced B-subunit is once again at *m*/*z* 7821.

**Fig. 12:**
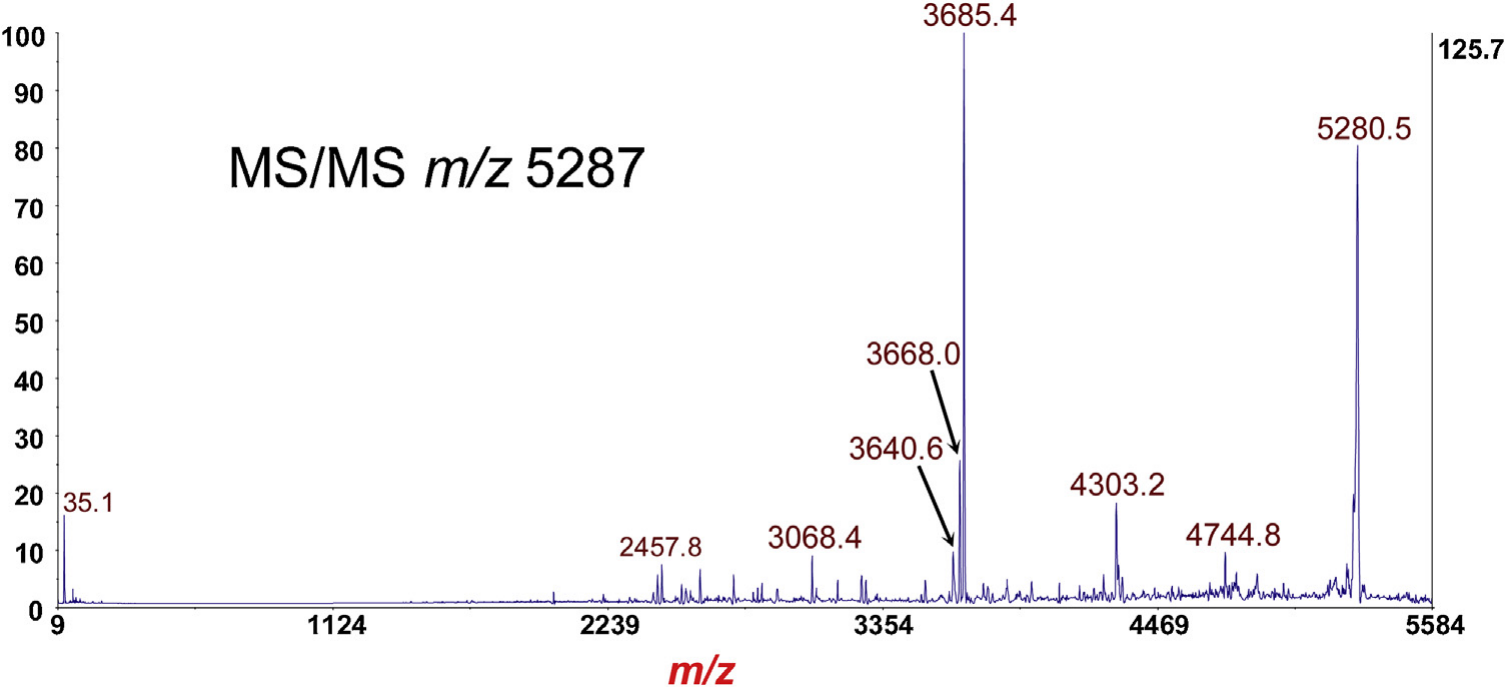
MS/MS-PSD of *m*/*z* at 5287 of the A2 fragment of the A-subunit from Fig. 11. Isolation mass window: −100 Da/+25 Da.

The method was validated using *Escherichia coli* O157:H7 strain EDL933 which was found to strongly induce Stx expression by culturing overnight on LBA supplemented by 20 ng/mL of ciprofloxacin. The following figures show a step-by-step progression of the analysis of this STEC strain. The figure captions summarize the data presented (Fig. 6, Fig. 7, Fig. 8, Fig. 9, Fig. 10, Fig. 11, Fig. 12).

## References

[1] Fagerquist C.K., Zaragoza W.J. (2016). Bacteriophage cell lysis of Shiga toxin-producing *Escherichia coli* for top-down proteomic identification of Shiga toxins 1 & 2 using matrix-assisted laser desorption/ionization tandem time-of-flight mass spectrometry. Rapid Commun. Mass Spectrom..

[2] Fagerquist C.K., Zaragoza W.J., Sultan O., Woo N., Quiñones B., Cooley M.B., Mandrell R.E. (2014). Top-down proteomic identification of Shiga toxin 2 subtypes from Shiga toxin-producing *Escherichia coli* by matrix-assisted laser desorption ionization-tandem time of flight mass spectrometry. Appl. Environ. Microbiol..

[3] Fagerquist C.K., Sultan O. (2011). Induction and identification of disulfide-intact and disulfide-reduced β-subunit of Shiga toxin 2 from *Escherichia coli* O157:H7 using MALDI-TOF-TOF-MS/MS and top-down proteomics. Analyst.

[4] Fagerquist C.K., Sultan O. (2010). Top-down proteomic identification of furin-cleaved α-subunit of Shiga toxin 2 from *Escherichia coli* O157:H7 using MALDI-TOF-TOF-MS/MS. J. Biomed. Biotechnol..

[5] Fagerquist C.K., Sultan O. (2012). A new calibrant for matrix-assisted laser desorption/ionization time-of-flight-time-of-flight post-source decay tandem mass spectrometry of non-digested proteins for top-down proteomic analysis. Rapid Commun. Mass Spectrom..

[6] Fagerquist C.K., Garbus B.R., Williams K.E., Bates A.H., Boyle S., Harden L.A. (2009). Web-based software for rapid top-down proteomic identification of protein biomarkers, with implications for bacterial identification. Appl. Environ. Microbiol..

[7] C.K. Fagerquist, L.A. Harden, B.R. Garbus, Rapid identification of proteins and their corresponding source organisms by gas phase fragmentation and identification of protein biomarkers. U.S. Patent: US8160819B2. 17 April 2012.

[8] Demirev P.A., Feldman A.B., Kowalski P., Lin J.S. (2005). Top-down proteomics for rapid identification of intact microorganisms. Anal. Chem..

[9] Fagerquist C.K., Garbus B.R., Miller W.G., Williams K.E., Yee E., Bates A.H., Boyle S., Harden L.A., Cooley M.B., Mandrell R.E. (2010). Rapid identification of protein biomarkers of *Escherichia coli* O157:H7 by matrix-assisted laser desorption ionization-time-of-flight-time-of-flight mass spectrometry and top-down proteomics. Anal. Chem..

